# Human Papillomavirus Infection, Infertility, and Assisted Reproductive Outcomes

**DOI:** 10.1155/2015/578423

**Published:** 2015-11-01

**Authors:** Nigel Pereira, Katherine M. Kucharczyk, Jaclyn L. Estes, Rachel S. Gerber, Jovana P. Lekovich, Rony T. Elias, Steven D. Spandorfer

**Affiliations:** ^1^The Ronald O. Perelman and Claudia Cohen Center for Reproductive Medicine, Weill Cornell Medical College, New York, NY 10021, USA; ^2^Department of Obstetrics and Gynecology, Weill Cornell Medical College, New York, NY 10021, USA

## Abstract

The human papillomavirus (HPV) is a sexually transmitted infection common among men and women across all geographic and socioeconomic subgroups worldwide. Recent evidence suggests that HPV infection may affect fertility and alter the efficacy of assisted reproductive technologies. In men, HPV infection can affect sperm parameters, specifically motility. HPV-infected sperm can transmit viral DNA to oocytes, which may be expressed in the developing blastocyst. HPV can increase trophoblastic apoptosis and reduce the endometrial implantation of trophoblastic cells, thus increasing the theoretical risk of miscarriage. Vertical transmission of HPV during pregnancy may be involved in the pathophysiology of preterm rupture of membranes and spontaneous preterm birth. In patients undergoing intrauterine insemination for idiopathic infertility, HPV infection confers a lower pregnancy rate. In contrast, the evidence regarding any detrimental impact of HPV infection on IVF outcomes is inconclusive. It has been suggested that vaccination could potentially counter HPV-related sperm impairment, trophoblastic apoptosis, and spontaneous miscarriages; however, these conclusions are based on in vitro studies rather than large-scale epidemiological studies. Improvement in the understanding of HPV sperm infection mechanisms and HPV transmission into the oocyte and developing blastocyst may help explain idiopathic causes of infertility and miscarriage.

## 1. Introduction

The human papillomavirus (HPV) is one of the most common sexually transmitted viruses worldwide [1, 2]. As recently as 1970, HPV was thought to be a single type that caused warty lesions at different tissue sites [3]. With the advent of recombinant DNA technology, however, it became evident that many different types of HPV existed, some of which were carcinogenic [3]. Current scientific literature tends to focus on HPV's association with cancer, particularly cervical cancer. However, infection with HPV is known to affect other aspects of human health as well. In fact, recent evidence suggests that HPV infection may affect fertility and alter the efficacy of assisted reproductive technologies [4, 5]. The current review appraises the recent medical literature pertaining to the epidemiology, immunobiology, and impact of HPV infection on normal reproductive function. Furthermore, the paper critically evaluates the current evidence related to HPV infection and fertility alteration, as well as its impact on assisted reproductive pregnancy rates.

## 2. Epidemiology

Despite licensure of HPV vaccines in more than half of the world's countries, the global HPV prevalence was estimated at 12% in 2012 [6, 7]. Most recent data indicate that 14 million people are newly infected each year in the United States, with a total of 79 million people currently affected [8]. The virus's continuing prevalence is partially attributed to inconsistent vaccination rates [9, 10]. The overall cost burden of preventing and treating HPV-associated disease in 2010 was estimated to be $8.0 billion [11].

HPV infection is generally transmitted by skin-to-skin contact and infects the epithelial cells in genital mucosa, oral mucosa, or skin [12, 13]. Most sexually active adults will acquire HPV in their lifetime [8], though it can occur at any age [14]. Some studies have shown a U-shaped curve with regards to HPV infection, meaning infection rates peak in women under the age of 30 and also in women aged 55–64 years [15]. In general, the risk of acquiring HPV increases with the number of recent and lifetime sexual partners [8, 16, 17]. Almost 40% of all women infected with human immunodeficiency virus (HIV) will also have coinfection with multiple HPV genotypes [18].

## 3. Immunobiology of HPV

HPV is a double-stranded nonenveloped DNA adenovirus, which belongs to a large family of more than 130 genotypes [19, 20]. The genome of the virus can be divided into three main domains: a noncoding upstream regulatory region of 1 kb in size; an early region with six genes, namely, E6, E7, E1, E2, E4, and E5; and a late region with two genes, L1 (major capsid protein) and L2 (minor capsid protein) [17]. These viruses are generally classified as either low-risk types that cause benign warts or high-risk types that are associated with cancers [3]. While HPV 6 and 11 are the most common low-risk types that cause anogenital warts, HPV 16 and 18 are the most common oncogenic or high-risk types, which are responsible for up to 70% of all cervical cancers worldwide [21, 22]. The different types of papilloma viruses exhibit characteristic tropism: cutaneotropic (HPV 1, 4, 5, 8, 41, 48, 60, 63, and 65) types are isolated in cutaneous and plantar warts, whereas mucosotropic (HPV 6, 11, 13, 18, 39 44, 55, 16, 31, 33, 35, 52, 58, 67, etc.) types are identified in benign and malignant lesions of the anogenital tract, oral cavity, oropharynx, and larynx.

The female genital tract faces high and frequent antigenic exposures [19]. Before reproductive age, numerous antigens are recognized as “self” or “own” commensal microbiota [19]. However, once sexual activity begins, the genital tract must deal with exposure to several other exogenous antigens, including those derived from the male reproductive tract [19]. From a biological perspective, HPV is a very successful pathogen; that is, it can induce chronic infections without any systemic symptoms, allowing the host to periodically shed large amounts of transmissible virus to naïve individuals [3]. The evasion of the immune system by HPV is primarily dependent on the differentiation of cell layers in the mucocutaneous epithelium [3]. Following microtrauma or abrasions to the epithelium, the basal cell layer is exposed and vulnerable to infection by HPV at low numbers [17]. Viral DNA replication ensues, increasing the number of genome copies to about 50–100 [17]. The dividing cells maintain copy numbers of the viral genome and at the same time tightly regulate the expression of the E6 and E7 proteins at very low levels [3, 17]. When the host cells stop dividing and begin to differentiate into the upper layers of the epithelium [3], massive upregulation and replication of the viral genome occur (1000 copies per cell) along with abundant expression of the E6 and E7 proteins [17]. In the superficial layers of the epithelium, the L1 and L2 proteins are expressed and many thousand copies of infectious viral particles are assembled and shed [17]. This infectious cycle is completed in 2-3 weeks [17]. A large body of evidence suggests that oncogenic properties of high-risk HPV are related to the E6 and E7 proteins and their individual roles in disrupting the normal cell cycle of the host [17, 19].

Most HPV infections are subclinical and many present unnoticeable or mild symptoms [3, 19]. The incubation period of HPV ranges from 3 weeks to 8 months [3, 17]. Approximately 10–30% of women have spontaneous regression of HPV infection in 3 months [17]; 90% of women are able to clear the HPV infection within 2 years [3, 19]. The clearance of such infections occurs due to the development of cell-mediated immunity, accompanied by seroconversion and the antibody to the L1 major capsid protein [17]. A subset of women (10–15%) do not mount a successful cell-mediated immune response and they remain persistently infected [23]. These women remain at risk for developing high-grade disease and possibly cancer [24].

## 4. HPV and Cancers

HPV has been shown to play a key role in the development of various cancers [25]. Approximately 26,000 new HPV-related cancers (17,000 in women and 9,000 in men) are diagnosed in the United States annually [26]. The role of high-risk HPV in the malignant progression of cervical disease has now been known for over 30 years [27]. More recently [28], high-risk HPV has also been associated with cancers in anatomic locations such as head and neck [29, 30], oropharynx [31], lung [32, 33], and bladder [34]. Recent population-based studies in the United States have shown that 96% of cervical cancers, 93% of anal cancers, 64% of vaginal cancers, 51% of vulvar cancers, 36% of penile cancers, and 63% of oropharyngeal cancers are attributable to HPV [8, 25].

## 5. HPV and Fertility

It is widely accepted that sexually transmitted infections such as* Chlamydia trachomatis, Neisseria gonorrhoeae,* and* Treponema pallidum* can lead to alterations in fertility or even infertility [4]. Reproductive alterations have also been associated with sexually transmitted viruses, including HIV, cytomegalovirus, and herpes simplex virus [34, 35]. Recent data have also suggested HPV as a fertility-altering agent [4, 36]. HPV infections can induce two different pathways: an infectious virion-producing pathway and a noninfectious cancer-producing pathway [37]. Evidence suggests that the former pathway may be involved in fertility alteration [37]. However, the role of HPV as a direct cause of infertility remains uncertain [38].

### 5.1. HPV and Sperm

It is well established that HPV infections in men can result in semen contamination [39–41]. Previous investigations have demonstrated the presence of HPV DNA and RNA in the penile shaft, urethra, epididymis, and testis [42, 43]. In one study of 111 men whose intimate female partners were infected with HPV, HPV DNA was detected in the semen of 23.4% of men [44]. It is thought that HPV binds to 2 distinct sites along the equatorial region of the spermatozoon's head [45–47]. The presence of glycosaminoglycans or other soluble factors on the surface of the spermatozoon is believed to mediate the interaction and binding between HPV and sperm [45–47]. Recent evidence suggests that the L1 HPV capsid protein and glycosaminoglycan syndecan-1 colocalize in the equatorial region of the sperm head [48].

### 5.2. Effect on Sperm Parameters

In general, sexually transmitted infections may affect semen quality by inducing orchitis, epididymitis, urethritis, or urethral stickiness [4, 47]. Yet the exact mechanisms by which HPV impairs sperm quality remain poorly understood. HPV infections in men can alter sperm motility [48, 49]. In one study, investigators found enhanced motility along with decreased velocity and amplitude of lateral head displacement [50]. An independent group's work [51] confirmed the findings of enhanced motility, progression, and velocity in HPV-exposed sperm. The majority of studies, however, indicate decreased sperm motility in HPV-infected men [36, 46, 52, 53]. These findings remain consistent among infertile males and sperm donors. HPV-related impairment in sperm motility is frequently found in men with idiopathic infertility compared to healthy fertile controls [54]. HPV is also known to increase sperm DNA fragmentation [55] and induce changes in semen pH [56]. Other semen parameters such as volume, viscosity, count, and morphology are not different in HPV-infected and in noninfected semen samples [47].

### 5.3. Transmission of HPV and Early Embryogenesis

Initial studies showed that sperm could carry exogenous HPV DNA and could potentially act as a vector that transmits HPV to sexual partners, as well as to a fetus through fertilized oocytes [39, 57–59]. In mouse models, HPV-infected sperm successfully fertilized oocytes [60], followed by subsequent gene expression in the inner cell mass and trophectoderm of early blastocysts [61]. Using the hamster egg-human sperm penetration test, investigators similarly demonstrated that human sperm could transfer the E6/E7 genes and the L1 major capsid protein to oocytes, with gene expression resulting in developing blastocysts [48]. Following expression of the E6/E7 genes, increased DNA fragmentation and trophoblast death were noted in blastocysts [62–64]. These findings were particularly associated with the HPV 16 subtype [63, 64]. The rate of HPV-related trophoblastic apoptosis seems to be related to the growth of the embryo; that is, the apoptosis rate is 3-fold and 5.8-fold greater at 3 and 12 days after fertilization, respectively [65]. It is important to note that the aforementioned findings are based on in vitro experiments in murine models, which may not necessarily reflect in vivo conditions in humans [36].

### 5.4. HPV and Early Pregnancy

In addition to its impact on trophoblastic growth, HPV seems to reduce the endometrial implantation of trophoblastic cells, thus increasing the theoretical risk of miscarriage [66–68]. In a study involving 108 patients with miscarriages, HPV 16 and 18 DNA was detected in 7.4% of all conceptuses tested [69]. In another study comparing 25 early miscarriages to 15 voluntary terminations of pregnancy, HPV E6/E7 sequences were detected in 60% of the former compared to 20% of the latter [70]. The finding that HPV DNA was detected more frequently in spontaneous miscarriages compared to voluntary abortions led to the consideration that HPV may be involved in the pathophysiology of early pregnancy loss [70]. Some studies have also indicated that exposure to HPV 6, 11, 16, or 18 in pregnancy can be associated with a 2.2% prevalence of major birth defects and a 1.5% risk of fetal death [68]. These findings, however, should be interpreted with caution, as they arise from retrospective or cross-sectional studies with small sample sizes. Contrary to these findings, at least 2 retrospective studies with larger study cohorts have shown that HPV infection in pregnancy may not confer a higher risk of miscarriage [71, 72].  Table 1 summarizes the findings of the studies pertaining to the effect of HPV on fertility.

## 6. HPV and Assisted Reproduction

### 6.1. Intrauterine Insemination

The prevalence of HPV sperm infection is approximately 2–31% in the general population, while the corresponding figure in men with unexplained infertility ranges between 10 and 35.7% [54]. In fact, some studies suggest that idiopathic asthenozoospermia may not have any risk factors except for the presence of HPV DNA [46, 47]. In a recent retrospective study of 590 women undergoing 1529 intrauterine insemination (IUI) cycles [73], the authors reported an 11% prevalence of HPV per IUI cycle. Furthermore, women with HPV infection were six times less likely to become pregnant (1.87%) compared to women without HPV infection (11.4%).

### 6.2. In Vitro Fertilization

Women with subfertility or infertility eligible for in vitro fertilization (IVF) are known to have almost twice the rate of HPV-related abnormal cervical cytology or high-grade cervical lesions compared to the general population [74–76]. It is therefore necessary to assess the impact of HPV on IVF outcome, if any. In one of the earliest studies evaluating the impact of HPV on IVF outcomes [5], the investigators detected HPV in 16% of their study cohort of 106 patients. Although no difference was found in the number of oocytes retrieved, number of embryos transferred, embryo quality, or spontaneous miscarriage rates, women with HPV infection had a lower pregnancy rate (23.5%) compared to women without HPV infection (57.0%). In another study of 199 infertile couples, 9.5%, 17.5%, and 4.5% of males, females, and both partners were found to be HPV positive, respectively [77]. The authors reported higher odds of spontaneous miscarriage (odds ratio: 4.20) among women with HPV infection, as well as in women whose male partners were HPV positive (odds ratio: 11.3). A different group of investigators noted lower birth rates in HPV positive women compared to negative controls, though these differences were not statistically different owing to a small sample size [78]. However, subsequent studies have shown conflicting results, with at least 3 studies showing no effect of HPV on clinical pregnancy and spontaneous miscarriage rates after IVF [38, 79, 80].  Table 2 summarizes the literature regarding the effect of HPV on assisted reproductive outcomes.

## 7. HPV and Adverse Pregnancy Outcomes

The detection of HPV in placental tissue suggests vertical transmission of HPV [59, 67]. In a study of 291 pregnant women of > 36 weeks of gestation, the rate of vertical transmission was estimated to be 18.2% [81]. Vertical transmission was often noted when an infant was delivered through an infected cervix; however, the absence of HPV infection in all infants at 6 months suggested temporary inoculation rather than a vertical infection [81]. Despite only transient detection of HPV DNA, there is speculation that HPV may play a possible role in adverse pregnancy outcomes. Recent evidence suggests that HPV, at least in part, is associated with preterm rupture of membranes [82, 83] and spontaneous preterm birth [84–86].

## 8. HPV Vaccination

Currently, the HPV vaccine is approved for prevention of genital warts, cervical dysplasia, and cervical cancer [3, 20]. Given the emerging association of HPV infection with other malignancies as well as fertility alteration, such a vaccine could help reduce HPV-related disease burden. The primary rationale for utilizing HPV vaccines would be to counter any HPV-related sperm impairment in couples with idiopathic infertility [66]. Furthermore, from a theoretical standpoint, the HPV vaccination could prevent HPV-related trophoblastic apoptosis and spontaneous miscarriages, thereby improving assisted reproductive outcomes [66]. HPV infection of sperm is also a large-scale problem for sperm donor banks [87, 88]. In the absence of effective sperm washing procedures that are able to eliminate HPV infection, male vaccination can be considered a possible strategy for the prevention of HPV-related sperm impairment in donors [66]. Although the aforementioned strategies seem reasonable, it must be noted that these suggestions are based on in vitro studies rather than large-scale epidemiological studies.

## 9. Conclusions

The human papillomavirus (HPV) is a ubiquitous sexually transmitted infection, which often goes undiagnosed. Nevertheless, its wide prevalence has helped uncover some of the negative ramifications of infection with the virus. Although HPV is most widely known for its link to various cancers, recent evidence also suggests an association with infertility and adverse pregnancy outcomes. Regarding fertility, HPV seems to affect both men and women—the virus can bind to the head of a spermatozoon and reduce sperm motility in men and may reduce the endometrial implantation of trophoblastic cells in women. Blastocysts that resulted from HPV-infected sperm were found to have expression of HPV-related genes with corresponding trophoblastic apoptosis. The role of HPV in the success of assisted reproduction is less clear-cut; several studies show a decreased pregnancy rate for intrauterine insemination and in vitro fertilization in women with HPV compared to controls, while other studies show no correlation. HPV has also been linked with preterm rupture of membranes, spontaneous preterm birth, and a potentially increased rate of early pregnancy loss. Given the wide impact of the virus on human health, vaccination of men and women will be vital to the reduction of disease burden on future patients.

## Figures and Tables

**Table 1:** Summary of literature pertaining to the effect of HPV on fertility.

Reference	Study type	Summary of findings
Effect on sperm parameters		
[47]	Systematic review	No effect on volume, viscosity, count, and morphology
[50]	Original research	Enhanced motility; decreased velocity and amplitude of HPV-exposed sperm
[51]	Original research	Enhanced motility, progression, and velocity in HPV-exposed sperm
[52, 53]	Original research	Decreased sperm motility in HPV-infected men
[54]	Systematic review	HPV-related impairment in sperm motility in men with idiopathic infertility
[55]	Original research	Increase in DNA fragmentation
[56]	Original research	Semen pH is borderline lower in HPV-positive men

Effect on early embryogenesis		
[62–64]	Original research	Increased DNA fragmentation and trophoblastic death in blastocysts

Effect on early pregnancy		
[66–68]	Original research	HPV reduces the endometrial implantation of trophoblastic cells
[69, 70]	Original research	HPV DNA is more frequently detected in spontaneous miscarriages compared to voluntary abortions
[71, 72]	Original research	HPV infection in pregnancy may not confer a higher risk of miscarriage

**Table 2:** Summary of literature regarding the effect of HPV on assisted reproductive outcomes.

Reference	Study type	Summary of findings
Intrauterine insemination		
[46, 47]	Original research Systematic review	HPV DNA may be associated with idiopathic asthenozoospermia
[73]	Original research	Women with HPV were six times less likely to become pregnant compared to women without the infection

In vitro fertilization		
[5]	Original research	Women with HPV infection had a lower pregnancy rate compared to women without the infection
[77]	Original research	Higher odds of spontaneous miscarriage among women with HPV infection, as well as in women whose male partners were HPV positive
[78]	Original research	No statistical difference in live birth rate when comparing HPV positive women compared to negative controls
[38, 79, 80]	Original research	No effect of HPV on clinical pregnancy and spontaneous miscarriage rates
